# Application of the Ballroom Aerobic Test protocol for assessing performance in standard-style dancesport athletes

**DOI:** 10.7717/peerj.20556

**Published:** 2026-01-13

**Authors:** Tamara Despot, Davor Plavec

**Affiliations:** 1 Faculty of Kinesiology, University Zagreb, Zagreb, Croatia; 2 Dance Studio ‘Dancespot’, Zagreb, Croatia; 3 Facility for Medical Care ‘Prima Nova’, Zagreb, Croatia; 4 Medical Faculty, Josip Juraj Strossmayer University of Osijek, Osijek, Croatia

**Keywords:** Aesthetic sports, The BAT protocol, Cardiorespiratory fitness, Effect size, Coefficient of variation, Typical error, Smallest worthwhile change

## Abstract

**Background:**

Although the existing field-based tests to assess functional capacity in dancesport have been developed, most of them have been applied to ballet or contemporary dancesport athletes at the individual level, while little evidence is available for dancesport couples in standard dance disciplines. Therefore, the main purpose of this study was to examine the test-retest reliability and usefulness of the progressive Ballroom Aerobic Test (BAT) protocol for assessing aerobic performance.

**Methods:**

Thirteen standard dancesport athletes (six pairs and one individual male participant; age: 20.1 ± 3.8 years; height: 175.0 ± 8.2 cm; weight: 64.3 ± 9.7 kg; males; age: 19.9 ± 3.7 years; height: 180.0 ± 4.7 cm; weight: 70.2 ± 8.7 kg; body mass index: 21.5 ± 1.8 kg/m2 training experience: 8.6 ± 5.1 years; females; age: 20.3 ± 4.0 years; height: 168.5 ± 6.5 cm; weight: 57.5 ± 5.5 kg; body mass index: 20.2 ± 1.3 kg/m^2^; training experience: 8.5 ± 3.4 years) took part in the study. All participants were members of certified dancesport clubs who competed at national and international levels. The BAT protocol consisted of five stages, each corresponding to one of five standard dancesport disciplines (English Waltz, Slow Foxtrot, Tango, Viennese Waltz, and Quickstep). A test-retest design, with one week between trials, was used to examine the reliability measures. Ventilatory and metabolic parameters were derived from MetaMax^®^ 3B, a reliable and valid portable gas analyzer. Reliability measures included Cohen D effect size (ES), intra-class correlation coefficient (ICC), coefficient of variation (CV), typical error (TE) and smallest worthwhile change (SWC).

**Results:**

No significant differences were observed between test and retest sessions (*P* > 0.05). ESs ranged from trivial to small (0.00–0.47) with ‘very good’ to ‘excellent’ reliability (ICC = 0.80–0.99; CV = 1.63–4.85%). The usefulness of the BAT protocol was generally rated as ‘ok’ to ‘good’ for speed (TE = 2.76; SWC = 3.20; <), absolute VO_2_ (TE = 0.14; SWC = 0.15; <), respiratory exchange ratio (RER; TE = 0.02; SWC = 0.02; =), tidal volume (VT = 0.06; SWC = 0.06; =), and dead space to tidal volume ratio (VD/VT; TE = 0.01; SWC = 0.01; =).

**Conclusion:**

Current findings suggest that the BAT protocol is a reliable and useful tool for assessing aerobic capacity in standard-style dancesport athletes. Moreover, the BAT protocol can accurately detect meaningful individual changes, particularly for speed (3.20 bpm), absolute VO_2_ (0.15 L*min^−1^), and VE (4.87 L). These results may assist athletes and strength and conditioning coaches in monitoring and tracking ‘real’ time changes to optimize the training process.

## Introduction

Dancesport performance has been categorized as a high-intensity intermittent exercise requiring high levels of physical fitness (Koutedakis & Jamurtas, 2004). In addition to technical requirements, the complexity of choreography makes physical preparedness just as important as skill development (Ngo et al., 2024), with upper body muscular and cardiovascular endurance identified as the best predictors of aesthetic performance (Angioi et al., 2009). Although dancesport is composed of many disciplines, physiological capabilities are crucial to sustaining the demanding daily schedules of training and competition performance. Evidence suggests that aerobic capacity is one of the key determinants of success in dancesport (Smol & Fredyk, 2012). For example, a study by Rodrigues-Krause, Krause & Reischak-Oliveira (2015) showed that dancers’ cardiorespiratory requirements during both dancesport classes and performances were equally important. However, aerobic capacity during rehearsals and/or classes exhibited lower values than during competition. This suggests that, despite being a crucial factor distinguishing successful from unsuccessful dancesport athletes (Malkogeorgos et al., 2013), the discrepancy between training and competition in dancesport athletes might limit the ability to improve or even maintain higher levels of cardiorespiratory physical fitness (Nam, Park & Kim, 2024). The ability to efficiently uptake, transport and utilize oxygen through physiological processes has been prioritized, as it directly influences technical achievements, performance quality, and career longevity (Angioi et al., 2012). However, both professional and student dancesport athletes tend to demonstrate lower maximal oxygen uptake (VO_2_max) compared to other athletes (Baldari & Guidetti, 2001; Tiemens et al., 2023). For instance, male dancesport athletes exhibit VO_2_max of 48.0 ml*kg^−1^*min^−1^, which is 20 to 50% lower than values reported in cyclic or complex team sport activities (Cohen et al., 1982; Koutedakis & Jamurtas, 2004). Another contributing factor to VO_2_max improvements in dance may be related to its small ability to stimulate positive cardiorespiratory adaptations (McCord, Nichols & Patterson, 1989). It has been documented that increases in aerobic capacity are not related to class work, but rather to the intensity and duration of performances (Wyon et al., 2004). Since the intensity of specific (dance movements) or general (circuit) training regimes needs to be between 70 and 80% of an individual’s VO_2_max and should last >20 min to enhance aerobic capacity, progressive dance protocols to stimulate cardiorespiratory development are warranted.

In dancesport, measuring aerobic capacity with objective tools (heart rate monitors, ergometry) is the cornerstone for planning training programs at the individual level (Tiemens et al., 2023). Because of complex movement patterns and often strenuous training and competition programs, energy pathways for both aerobic and anaerobic requirements should be considered equally important (Maciejczyk & Feć, 2013). On the other hand, literature suggests that physical performance can be a useful indicator of physiological tremor and fatigue (Kruusamäe, Maasalu & Jürimäe, 2016; Mazur-Rózycka et al., 2023), adding its practical ability to detect the level of endurance and deteriorating effects of training and competition on dancesport performance (Ngo et al., 2024). The necessity of determining an individual’s cardiovascular and respiratory capabilities may be helpful in dosing the training load and primarily targeting positive aerobic output during preparatory period. On the other hand, examining ‘maximal’ aerobic levels in dancesport is quite difficult to achieve, because the nature of dance is non-competitive, and the intensity is often set by a choreographer (Chatfield et al., 1990). Unfortunately, well-established objective methods to assess aerobic performance, such as running- or cycling-based ergometers, are expensive, difficult to administer and do not mimic complex dancesport movements. Moreover, dancers may have biomechanical limitations during running or walking, due to their limited dorsiflexion movements in the ankle and a highly developed turn out of the feet, which may cause stress on the knee and hip joints (Russell, 2013). Because of these limitations, previous studies have attempted to develop on-field aerobic tests for dancesport. According to a systematic review by Tiemens et al. (2023), the most common cardiorespiratory fitness test protocols in dancesport include the Aerobic Power Index (API) (Wallman et al., 2003), the Ballet-specific Aerobic Fitness Test (B-DAFT) (Twitchett et al., 2011), the Dance Aerobic Fitness Test (DAFT) (Wyon et al., 2023), the High-Intensity Dance Performance Fitness Test (HIDT) (Redding et al., 2009), and the Seifert Assessment of Functional Capacity for Dancers (SAFD) (Seifert et al., 2021).

Despite the fact that all tests exhibited ‘good’ to ‘very good’ test-retest reliability properties, findings from these studies cannot be generalized to other dancesport disciplines. Most studies recruited ballet (Twitchett et al., 2011), or contemporary (Wyon et al., 2004) dancesport athletes, whose training routines and movement patterns differ from those of standard dancesport practitioners (Ngo et al., 2024). Standard male (59.6 ml*kg^−1^*min^−1^) and female dancers (51.2 ml*kg^−1^*min^−1^) tend to have higher average VO_2_max values (Liiv et al., 2014), compared to ballet or contemporary dancesport athletes (Wyon et al., 2004; Twitchett et al., 2011). Moreover, VO_2_max has been the most commonly used variable to assess aerobic capacity (Wallman et al., 2003; Redding et al., 2009; Twitchett et al., 2011; Wyon et al., 2023), while data regarding speed, ventilatory and metabolic parameters have been rarely investigated. Finally, available field-based tests have been developed at the individual level, and to the best of the authors’ knowledge, the test-retest properties have not been evaluated simultaneously in dancesport couples. These discrepancies between different dancesport disciplines, type of dancesport athletes and physiological characteristics may affect the reliability properties of a given test. Notably, for the abovementioned tests assessing aerobic performance, neither typical error (TE) nor smallest worthwhile change (SWC) were calculated, limiting their usefulness in real-time settings. TE indicates the random error associated with a measurement protocol, while SWC represents the smallest worthwhile change that is practically relevant to an athlete (Hopkins, 2004; Hopkins, 2007). Both measures allow us to determine if observed performance changes in a certain variable are greater than the TE and the SWC, denoting statistical significance and practical importance.

The reliability and usefulness of a newly developed progressive dance test to assess aerobic capacity would be of great importance for standard-style dancesport athletes and their strength and conditioning coaches, allowing them to monitor and track training intensity and duration for maximum efficiency. If results showed appropriate reliability statistics with confirmatory usefulness, the new progressive test would better reflect the true energetic state of dancesport athletes in both training and competitive conditions. Based on the shortcomings of previously established dancesport tests, the main purpose of this study was to examine the test-retest reliability of the Ballroom Aerobic Test (BAT) for assessing aerobic capacity in dancesport athletes competing in standard dancesport disciplines. The second purpose was to determine its usefulness in accordance with the noise of the test results and measurement uncertainty using the magnitude of the SWC. We hypothesized that the BAT test would yield ‘good’ reliability and could detect meaningful changes in performance.

## Materials and Methods

### Experimental approach

This observational, within-subject, test-retest sub-study was conducted to examine the reliability properties and usefulness of the BAT in standard dancesport athletes. Portions of this text were previously published as part of a study (Despot & Plavec, 2025). In brief, the study was part of a project titled ‘Construction and Validation of a Test Protocol to Assess Aerobic Capacity in Dancesport Athletes from Standard Dance Styles.’ (Despot & Plavec, 2025). The project was divided into four segments to examine: (i) reliability and usefulness; (ii) validity; (iii) sensitivity; and (iv) pragmatic validity of the BAT. For the purpose of this study, the BAT protocol was performed on two separate occasions, one week apart. The testing procedure was initiated during the preparatory period of dancesport athletes. Measurements were performed by the same experienced researcher to minimize possible measurement error. During both trials, the participants wore their standard dance equipment (light T-shirt, tights, dance shoes). The air temperature conditions of the indoor facility were controlled to be between 22 °C and 24 °C with the humidity of ≈ 55%.

### Study participants

Thirteen advanced standard dancesport athletes (six pairs and one male participant; age: 20.1 ± 3.8 years; height: 175.0 ± 8.2 cm; weight: 64.3 ± 9.7 kg) with >8 years of training and competing experience participated in the study. As mentioned in a previous study (Despot & Plavec, 2025), all participants were members of certified dancesport clubs who competed at national and international levels. The inclusion criteria to enter the study were: (i) being free from any kind of injury, acute or chronic illness and disease, as confirmed by a certified dancesport association doctor; (ii) age range between 16 and 35 years; and (iii) completing both measurements for reliability and usefulness properties of the BAT protocol. The *a-priori* power analysis calculated by the G*Power software ver. 3.1.9.7 (Kang, 2021) showed that by setting the input parameters of a two-tailed *α* < 0.05, a minimum required correlation between two occasions at *r* > 0.69 (Buchheit et al., 2011), and statistical power of 1 − *β* = 0.80, the required total sample size was *n* = 11. Before the study began, all participants had signed a written informed consent to participate in the study and to allow their data to be used solely for scientific purposes. All procedures in the study were anonymous and conducted in accordance with the Declaration of Helsinki (World Medical Association, 2013). The Ethical Committee of the Faculty of Kinesiology approved the study on 29 April 2020 (ethical code number: 77/2020).

### Study variables

The same methodology to examine the psychometric properties of the BAT protocol has been published elsewhere (Despot & Plavec, 2025). To examine the reliability properties of the BAT protocol, we used the MetaMax^®^ 3B (CORTEX Biophysik GmbH, Leipzig, Germany), a reliable and valid portable breath-by-breath ventilatory and metabolic measurement system specifically designed for field- and laboratory-based testing (Macfarlane & Wong, 2012). The device consists of two parts designed to be worn on the chest. By using an electrochemical cell and an infrared analyzer, the MetaMax^®^ 3B was able to calculate O_2_ and CO_2_ concentrations based on standard metabolic equations (Wasserman et al., 1999). Following the manufacturer’s recommendations and previous studies (Macfarlane & Wong, 2012), the system was turned on for at least 20 min and calibrated prior to testing. The calibration process included adjusting the gas analyzers by using reference gas values of O_2_ (14.97%), CO_2_ (4.96%) and N_2_ (± 0.02%) and volume with a standardized 3-L syringe (5530 series, Hans Rudolph, Inc., Shawnee, KS, USA). The MetaMax^®^ 3B software generated data regarding absolute (L*min^−1^) and relative VO_2_max (ml*kg^−1^*min^−1^) and associated variables including speed (bpm), respiratory exchange ratio of CO_2_ produced to O_2_ consumed during metabolism (RER), expiratory ventilation as the total volume of air inhaled or exhaled per min (VE; L*min^−1^), tidal volume as the volume inhaled or exhaled during a normal breath (VT; L), breathing frequency (BF; bpm), VO_2_ andBF norms calculated from referent values provided by the MetaMax^®^ 3B (%), ventilatory equivalents for O_2_ (VE/VO_2_) and CO_2_ (VE/VCO_2_) as determinants of breathing efficiency, and dead space to tidal volume ratio (VD/VT), which indicated the air that failed to participate in O_2_ and CO_2_ exchange process during breathing.

### The BAT protocol

Of note, portions of this text were previously published as part of a study conducted by Despot & Plavec (2025). To assess the level of aerobic capacity of dancesport athletes, we constructed a field-based BAT protocol. The protocol was standardized across five standard dancesport disciplines according to the World DanceSport Federation (Premelč et al., 2019) in the following order: (i) English waltz; (ii) Slow foxtrot; (iii) Tango; (iv) Viennese waltz; and (v) Quickstep. Each dancesport discipline included multiple levels of dancing that were progressively linked, where the speed of dancing (tempo) was defined both by individual beats per minute (bpm), and by beat (bars per minute–BPM) which denote rhythm. The English waltz consisted of six levels, danced in 3/4 time in the range of 25–37 BPM or 75–110 bpm. The Slow foxtrot included five levels, danced in 4/4 time in the range of 29–36 BPM or 117–145 bpm. The Tango had five levels, danced in 4/4 time in the range of 38–45 BPM or 152–180 bpm. The Viennese waltz was performed through three levels, danced in 3/4 time in the range of 62–67 BPM or 187–201 bpm. Finally, the Quickstep consisted of six levels, danced in 4/4 time in the range of 52–60 BPM or 208–243 bpm. At each level, dancesport couples had to perform standard dance figures for 30 s at a prescribed tempo without accompanying music (Table 1). We did not include music, as studies have shown that it may significantly impact both physical and psychological performance, by increasing the ratings of pleasure and emotional arousal during more complex musical pieces (Salimpoor et al., 2009), affecting the autonomic nervous system (Lundqvist et al., 2009), and altering the rhythm of movements (Hu, Li & Liu, 2022), all of which are contributing factors to dancesport performance. The speed expressed in beats progressively increased throughout the protocol by seven BPM. The initial speed of the dancesport protocol was set at 75 bpm. Participants danced to a predefined dance tempo and rhythm using a metronome. The elements of each dancesport discipline were basic and easy to perform, while dancesport couples were free to create their own choreography. The duration of the BAT protocol was cumulatively added up and each pair danced until exhaustion.

**Table 1:** The BAT dance protocol for every dance style.

**Dance styles**	**Stage 1**	**Stage 2**	**Stage 3**	**Stage 4**	**Stage 5**	**Stage 6**
*English waltz*						
Beats	75	82	89	96	103	110
Time	30 s	60 s	1.5 min	2 min	2.5 min	3 min
*Slow fox*						
Beats	117	124	131	138	145	
Time	3.5 min	4.0 min	4.5 min	5.0 min	5.5 min	
*Tango*						
Beats	152	159	166	173	180	
Time	6.0 min	6.5 min	7.0 min	7.5 min	8.0 min	
*Viennese waltz*						
Beats	187	194	201			
Time	8.5 min	9.0 min	9.5 min			
*Quick step*						
Beats	208	215	222	229	236	243
Time	10.0 min	10.5 min	11.0 min	11.5 min	12.0 min	12.5 min

### Data analysis

Shapiro–Wilk’s (S–W) test was used to assess data normality. Data were checked for critical *W* values, Q–Q plot inspection, and the homogeneity of the variance was confirmed using the Leven test. If the calculated *W* value was larger than the tabulated *W* value, this denoted that we did not reject the null hypothesis. All variables included in further analysis were assumed to follow a hypothetical normal distribution, so data were presented as means and standard deviations (SD) with 90% confidence interval limits (90% CI). The magnitude of change between the two measurements (T0 and T1) were examined using a standardized mean difference with the following effect size (ES) proposed by Hopkins, Schabort & Hawley (2001): trivial (ES < 0.2), small (0.2 < ES < 0.5), moderate (0.5 < ES < 0.8), large (0.8 < ES < 1.6), and very large (ES > 1.6). The reliability statistics between *pre-* and *post-* measurements were determined using three parameters: (i) intraclass correlation coefficient (ICC), (ii) typical error (TE) expressed as coefficient of variation (CV%), and (iii) smallest worthwhile change (SWC), calculated using an Excel spread sheet provided by Hopkins (2007). Based on the 90% CI of the ICC estimate provided by Koo & Li (2016), the ICC strength was described as ‘poor’ (ICC ≤ 0.5), ‘moderate’ (0.5 < ICC ≤ 0.75), ‘good’ (0.75 < ICC ≤ 0.9), and ‘excellent’ (ICC > 0.9). The CV% was calculated as the ratio of the SD to the mean and multiplied by 100, while the SWC as the between-subject SD multiplied by 0.2, according to ES. According to Hopkins (2004), the usefulness of a given variable was determined by comparing TE and SWC, and categorized as ‘marginal’ (TE > SWC), ‘ok’ (TE = SWC), and ‘good’ (TE < SWC). The criteria for considering reliability statistics as ‘adequate’ was CV < 5% and ICC > 0.69 (Buchheit et al., 2011). All statistical analyses were performed in SPSS v27.0 software (IBM, Armonk, NY, USA) with an alpha level set a priori at *p* < 0.05 to denote statistical significance.

## Results

Basic descriptive statistics of the study participants are presented in Table 2. Men were significantly taller and heavier compared to women. However, no significant differences were observed in age (*t*–value = 1.026, ES = 0.10), BMI (*t*–value = 1.374, ES = 0.72) and training experience (*t*–value = 0.029, ES = 0.02).

Table 3 presents the reliability statistics of aerobic capacity measures obtained by MetaMax^®^ 3B portable spiroergometric device. Test–retest data showed no significant *pre–post* differences (*P* > 0.05) in speed (pre = 220.54  ± 16.19 bpm; post = 214.92 ± 15.76 bpm), absolute VO_2_ (pre = 3.16 ± 0.63 L*min^−1^; post = 3.06 ± 0.67 L*min^−1^), relative VO_2_ (pre = 49.22 ± 5.84 ml*kg^−1^*min^−1^; post = 47.33 ± 6.28 ml*kg^−1^*min^−1^), RER (pre = 1.02 ± 0.05; post = 1.02 ± 0.05), VE (pre = 102.95 ± 25.09 L*min^−1^; post = 98.09 ± 23.53 L*min^−1^), VT (pre = 1.88 ± 0.29 L; post = 1.78 ± 0.29 L), BF (pre = 55.08 ± 9.18 bpm; post = 54.85 ± 8.46 bpm), VO_2_–norm (pre = 117.54 ± 18.36%; post = 112.00 ± 18.27%), BF–norm (pre = 32.31 ± 9.58%; post = 33.62 ± 9.62%), VO/VO_2_ (pre = 30.87 ± 2.84; post = 29.82 ± 2.34), VO/VCO_2_ (pre = 30.27 ± 2.84; post = 30.64 ± 3.44) and VD/VT (pre = 0.14 ± 0.02; post = 0.14 ± 0.01).

## Discussion

The main purpose of his study was to examine test-retest reliability properties and usefulness of the progressive BAT protocol for assessing aerobic capacity in athletes of standard dancesport disciplines. Findings suggest that the BAT protocol is a reliable new method based on objectively measured ventilatory and metabolic outcomes derived from the the MetaMax^®^ 3B portable device. In general, trivial to small ESs were observed between two occasions. The proposed criteria for ‘adequate’ reliability were met, with CV values for all the study variables < 5% (1.63 ≤ CV ≤ 4.85) and ICC values > 0.69 (0.80–0.99). The usefulness analysis of the BAT showed that the majority of variables were rated as ‘ok’ or ‘good’, indicating that the test is sufficiently sensitive to detect the smallest meaningful change.

**Table 2:** Basic descriptive statistics of the study participants.

**Study variables**	**Total (*n* = 13)**	**Men (*n* = 7)**	**Women (*n* = 6)**	***P*** ^*^
	Mean ± SD	Mean ± SD	Mean ± SD	
Age (years)	20.1 ± 3.8	20.3 ± 4.1	19.9 ± 3.5	0.254
Height (cm)	175.0 ± 8.2	180.6 ± 4.7	168.5 ± 6.5	0.003
Weight (kg)	64.3 ± 9.7	70.2 ± 8.7	57.5 ± 5.5	0.010
BMI (kg/m^2^)	20.9 ± 1.7	21.5 ± 1.8	20.2 ± 1.3	0.197
Experience (years)	8.5 ± 4.2	8.6 ± 5.1	8.5 ± 3.4	0.977

**Notes.**

* denotes significant differences between men and women (*P* < 0.05).

**Table 3:** Reliability measures for the MetaMax^®^ 3B aerobic outcomes.

	**ES**	**Mean diff. (90% CI)**	**ICC** **(90% CI)**	**CV%** **(90% CI)**	**TE** **(90% CI)**	**SWC** **(90% CI)**	**Rating**
**Speed** **(bpm)**	0.35 (small)	−5.62 (−7.55; −3.68)	0.98 (0.94; 0.99)	1.83 (1.10; 2.48)	2.76 (2.09; 4.19)	3.20 (2.41; 4.84)	Good
**Absolute VO**_**2**_ (L*min ^−1^ **)**	0.15 (trivial)	−0.1 (−0.20; −0.01)	0.96 (0.90; 0.99)	4.40 (2.70; 6.00)	0.14 (0.10; 0.21)	0.15 (0.10; 0.20)	Good
**Relative VO**_**2**_ (ml*kg^−1^*min^−1^)	0.31 (small)	−1.90 (−3.40; −0.40)	0.89 (0.71; 0.96)	4.52 (2.90; 6.00)	2.14 (1.61; 3.24)	1.21 (0.92; 1.85)	Marginal
**RER**	0.00 (trivial)	0.00 (−0.02; 0.01)	0.86 (0.67; 0.95)	1.63 (1.00; 2.30)	0.02 (0.02; 0.03)	0.02 (0.01; 0.03)	Ok
**VE** (L*min ^−1^ **)**	0.20 (small)	−4.85 (−8.07; −1.63)	0.97 (0.92; 0.99)	4.85 (3.34; 6.40)	4.61 (3.48; 6.98)	4.87 (3.68; 7.37)	Good
**VT** **(L)**	0.33 (small)	−0.09 (−0.14; −0.05)	0.96 (0.91; 0.99)	4.32 (3.20; 5.64)	0.06 (0.05; 0.09)	0.06 (0.04; 0.09)	Ok
**BF** **(bpm)**	0.03 (trivial)	−0.23 (−2.41; 1.94)	0.90 (0.74; 0.96)	4.57 (2.99; 6.68)	3.11 (2.35; 4.71)	1.77 (1.33; 2.68)	Marginal
**VO**_**2**_ **–norm** **(%)**	0.30 (small)	−5.54 (−7.68; −3.40)	0.98 (0.94; 0.99)	3.91 (2.80; 5.00)	3.06 (2.31; 4.64)	3.66 (2.77; 5.55)	Good
**BF–norm** **(%)**	0.14 (trivial)	1.31 (0.40; 2.22)	0.99 (0.96; 0.99)	4.71 (3.00; 6.26)	1.30 (0.98; 1.97)	1.92 (1.45; 2.91)	Good
**VE/VO**_**2**_	0.47 (small)	−1.05 (−1.74; −0.37)	0.86 (0.66; 0.94)	3.62 (2.78; 4.38)	0.98 (0.74; 1.49)	0.48 (0.36; 0.72)	Marginal
**VE/VCO**_**2**_	0.10 (trivial)	0.37 (−0.69; 1.43)	0.80 (0.54; 0.92)	3.83 (2.56; 5.40)	1.52 (1.15; 2.30)	0.63 (0.48; 0.96)	Marginal
**VD/VT**	0.00 (trivial)	0.00 (0.00; 0.01)	0.82 (0.58; 0.83)	4.40 (2.44; 6.38)	0.01 (0.01; 0.01)	0.01 (0.00; 0.02)	Ok

**Notes.**

ES effect size ICC intraclass correlation coefficient CV coefficient of variation TE typical error SWC smallest worthwhile change CI confidence intervals

Results from this study were considered in the context of previous studies aiming to develop and investigate the reliability statistics of field-based dancesport tests for measuring aerobic capacity (Wallman et al., 2003; Redding et al., 2009; Twitchett et al., 2011; Seifert et al., 2021; Wyon et al., 2023). In the study by Wallman et al. (2003), the API test showed ‘good’ test-retest reliability for aerobic output (ICC = 0.98; mean diff. = 0.02 ± 0.16 W*kg^−1^; CV = 3.87%), rate of perceived exertion (RPE; ICC = 0.94, mean diff. = 0.05 ± 2.10 a.u.; CV = 6.74%) and VO_2_max (ICC = 0.98; mean diff. = 0.41 ±2.13 ml*kg^−1^*min^−1^; CV = 4.63%). In another reliability study, the B-DAFT dancesport protocol produced an almost perfect correlation between the two trials (*r* = 0.998) with the 95% limits of agreement calculated from the Bland-Altman plots to be ± 6.2 ml*kg^−1^*min^−1^ (Twitchett et al., 2011). Redding et al. (2009) reported somewhat poorer agreement values for the HIDT, ranging from 0.80 between bouts of each trial and 0.82 between the four trials for maximal heart rate (HRmax), VO_2_ and blood lactate. A 3-min stage SAFD test for collegiate dancers exhibited moderated to excellent reliability for time (ICC = 0.85), VO_2_peak (ICC = 0.92), RER (ICC = 0.50), HR (ICC = 0.86), blood lactate (ICC = 0.52) and RPE (ICC = 0.80) with no significant differences between the two trials (Seifert et al., 2021). In current dancesport practice, the most widely used tool to assess aerobic capacity has been the DAFT (Wyon et al., 2023), a 4-min five stage test with a 5 ml*kg^−1^*min^−1^ bandwidth within each stage. Although the authors failed to provide the ICCs for VO_2_max and HRmax as the outcome variables of the telemetric gas analyzer (COSMED K4 b^2^, Italy), the CV for VO_2_max and HRmax ranged from 1.4 to 6.0% and 1.5 to 3.5% between stages, indicating adequate reliability (Wyon et al., 2023). Indeed, VO_2_ is the most common functional parameter for assessing consumption in dancesport athletes (Tiemens et al., 2023). Thus, our findings indicated that the ICCs for absolute and relative VO_2_ were 0.96 and 0.89, which is in line with the abovementioned evidence. However, we noted that the CV was <5%, which was somewhat lower than the data reported for the DAFT test. Although some studies have suggested that the guidelines for reliability statistics are ICC > 0.80, moderate CV of ≤ 10%, and small ES (<0.60) (Atkinson & Nevill, 1998; Hopkins, 2000), we additionally strengthened the inclusion criteria to insure the test is reliable and pragmatic in real-time settings. Thus, our newly developed BAT protocol appears to be a reliable measure for testing aerobic capacity and its components.

Data from this study also showed that the BAT protocol yielded appropriate sensitivity to detect meaningful changes following performance. Unfortunately, this is the first study to have calculated TE and SWC. When observing the findings in Table 3, ‘ok’ to ‘good’ usefulness was presented for speed, absolute VO_2_, RER, VE, VT, VO_2_ and BF norms and VD/VT, while relative VO_2_, BF, VE/VO_2_ and VE/VCO_2_ were deemed ‘marginally’ useful, as the TE was larger than the SWC. Changes in relative VO_2_ (1.21 ml*kg^−1^*min^−1^), BF (1.77 bpm), VE/VO_2_ (0.48) and VE/VCO_2_ (0.63) should be interpreted with caution, as the TE surpassed the SWC for capturing individual changes. Nevertheless, a higher proportion of variables were rated as ‘ok’ or ‘good’, confirming the BAT protocol as a useful test for aerobic performance monitoring. Although this study aimed to examine the test-retest reliability and usefulness of the BAT protocol, it is not without limitations. Sensitivity analysis calculated from the G*Power indicated that the appropriate sample size to detect ICC > 0.69 between the two occasions was *n* = 11, but we cannot exclude the possibility of insufficient statistical power. Additionally, training experience in dance ranged from 6 to 20 years, increasing the heterogeneity at both individual and dancesport couple levels. Because of the small sample size, we were unable to examine the differences based on experience.

Although dancesport is considered a high-intensity intermittent exercise (Koutedakis & Jamurtas, 2004; Rodrigues-Krause, Krause & Reischak-Oliveira, 2015), dancesport athletes are regarded as a ‘risky’ group of individuals with low level of cardiorespiratory fitness (Tiemens et al., 2023). Evidence regarding the development of aerobic capacity has consistently shown that none of their energy systems have been overly developed (Wyon, 2005). Another problem may be attributed to different energy requirements during the rehearsal and performance period, where the performance period is often accompanied by higher aerobic energy demands and more intense figure routines. Therefore, an advantage of the BAT protocol is that it simulates classical movements from five dancesport disciplines and mimics competition conditions. The current findings have provided evidence of its reliability properties and offer guidelines for the detection of meaningful changes in ventilatory and metabolic parameters. The authors suggest that this test can be implemented within the dancesport training program in preparatory and competitive periods, as it provides important information about changes and usefulness of aerobic performance variables. In terms of its’ ‘real’ applicability during training and choreography-based stimuli and the associations with long-term cardiovascular adaptations, the findings of the BAT protocol could be useful in enhancing both ventilatory and metabolic parameters related to dancesport activities. From a physiological perspective, the newly established dancesport protocol adequately addresses the most important factors of aerobic capacity in dancesport athletes, including VO_2_max. By following the BAT protocol, which consists of gradual increase of exercise intensity and duration every 30 s, positive changes in physiological abilities may be attributed to enhanced vascular tone and parasympathetic modulation of autonomic nervous system (Tomlinson & Naemi, 2025). These beneficial adaptations may contribute to faster recovery and less energy requirements for the same amount of activity, which leads to more sustainable sports performance during training or competition (Tao et al., 2022).

Although we obtained promising results demonstrating adequate reliability and usefulness of the BAT protocol, we cannot exclude a few potential limitations. Despite using calculator software with available input to calculate the appropriate sample size (for reference, please see the ‘Study participants’ section), a small sample of *n* = 13 might not be sensitive enough to capture the ‘true’ reliability and usefulness properties of the BAT protocol. This implies that the findings obtained in this sample may not be generalizable to other dancesport athletes from different disciplines and techniques. However, test-retest data indicated extremely high reliability and ability to detect meaningful TE and SWC. Nevertheless, this approach should be tested in other groups of dancesport athletes to confirm whether the BAT protocol yields satisfactory properties (Despot & Plavec, 2025).

## Conclusion

In summary, the BAT protocol is a reliable and useful progressive test for measuring aerobic capacity in standard discipline dancesport athletes. The test is suitable for administering in large groups indoors and can be completed in a relatively short period of time of ≈ 12–15 min. The characteristics through five stages which linearly progress by seven BPM and the duration of 30 s at each stage make this test easy to administer among dancesport couples while mimicking competition conditions. Moreover, by combining several dancesport disciplines (English waltz, Slow foxtrot, Tango, Viennese waltz, and Quickstep), coaches and their teams may be able to monitor and track ‘meaningful’ changes in response to training and/or detraining periods. It should be highlighted that 30-second intervals are capable of accurately capturing small ventilatory and metabolic differences derived from the portable MetaMax^®^ 3B gas analyzer. From a practical perspective, the BAT protocol is able to detect changes as low as 0.15 L*min^−1^ or 1.21 ml*kg^−1^*min^−1^ for absolute and relative VO_2_, providing a useful basis for planning and programming the training process during preparatory and competitive periods.

##  Supplemental Information

10.7717/peerj.20556/supp-1 Supplemental Information 1 Raw data

## Additional Information and Declarations

### Data Availability

The following information was supplied regarding data availability: The raw measurement is available in the Supplementary File.
